# Cellular Uptake of Tile-Assembled DNA Nanotubes

**DOI:** 10.3390/nano5010047

**Published:** 2014-12-30

**Authors:** Samet Kocabey, Hanna Meinl, Iain S. MacPherson, Valentina Cassinelli, Antonio Manetto, Simon Rothenfusser, Tim Liedl, Felix S. Lichtenegger

**Affiliations:** 1 Faculty of Physics and Center for Nanoscience, Ludwig-Maximilians University, Munich 80799, Germany; E-Mails: samet.kocabey@physik.lmu.de (S.K.); iainmacpherson@gmail.com (I.S.M.); tim.liedl@physik.lmu.de (T.L.); 2 Division of Clinical Pharmacology, Department of Internal Medicine IV, Klinikum der Universität München, Munich 80336, Germany; E-Mails: hanna.meinl@outlook.com (H.M.); simon.rothenfusser@med.uni-muenchen.de (S.R.); 3 Baseclick GmbH, Tutzing 82327, Germany; E-Mails: v.cassinelli@baseclick.eu (V.C.); a.manetto@baseclick.eu (A.M.); 4 Department of Internal Medicine III, Klinikum der Universität München, Munich 81377, Germany

**Keywords:** DNA nanotechnology, DNA tile, siRNA delivery, stability, folate, cation

## Abstract

DNA-based nanostructures have received great attention as molecular vehicles for cellular delivery of biomolecules and cancer drugs. Here, we report on the cellular uptake of tubule-like DNA tile-assembled nanostructures 27 nm in length and 8 nm in diameter that carry siRNA molecules, folic acid and fluorescent dyes. In our observations, the DNA structures are delivered to the endosome and do not reach the cytosol of the *GFP*-expressing HeLa cells that were used in the experiments. Consistent with this observation, no elevated silencing of the *GFP* gene could be detected. Furthermore, the presence of up to six molecules of folic acid on the carrier surface did not alter the uptake behavior and gene silencing. We further observed several challenges that have to be considered when performing *in vitro* and *in vivo* experiments with DNA structures: (i) DNA tile tubes consisting of 42 nt-long oligonucleotides and carrying single- or double-stranded extensions degrade within one hour in cell medium at 37 °C, while the same tubes without extensions are stable for up to eight hours. The degradation is caused mainly by the low concentration of divalent ions in the media. The lifetime in cell medium can be increased drastically by employing DNA tiles that are 84 nt long. (ii) Dyes may get cleaved from the oligonucleotides and then accumulate inside the cell close to the mitochondria, which can lead to misinterpretation of data generated by flow cytometry and fluorescence microscopy. (iii) Single-stranded DNA carrying fluorescent dyes are internalized at similar levels as the DNA tile-assembled tubes used here.

## 1. Introduction

Therapeutic agents must overcome multiple barriers to reach their target [1,2]. For example, siRNAs have to reach the target tissue, enter the cells, be released from the endosomal compartment and, finally, silence the target gene via the RISC complex [3]. Up to now, researchers have developed a variety of nanoparticle carrier systems to overcome these barriers, such as polymers [4], liposomes [5] or conjugates [6], with various levels of efficiency and toxicity. Most recently, with improvements in the DNA nanotechnology field, DNA-based nanostructures were developed as carrier systems for a variety of active components, including siRNAs [7], antibodies [8], immunostimulants [9,10] and cancer drugs [11,12]. DNA nanostructures are promising for delivery applications because they can be easily modified with a variety of (bio)chemical moieties for targeting purposes at nanoscale precision; they are monodisperse with well-defined sizes and are non-cytotoxic [10,13,14,15,16,17,18]. To date, several groups have investigated the targeted delivery of DNA-based nanostructures using different targeting agents, such as cell penetrating peptides or small molecules. Among them, folate is a commonly-used molecule, due to the high expression of its receptors on certain cancer cells. Efficient folate-mediated uptake has been demonstrated using various DNA-based structures, such as DNA nanotubes built from a single palindromic DNA strand [19] or Y-shaped DNA nanostructures prepared by rolling circle amplification [20]. Although the DNA-based nanostructures are promising for targeted delivery applications, as exemplified above, the stability of these structures at 37 °C in blood or tissue is one of the main issues to be considered. In a recent study, the stability of a variety of DNA origami structures with different designs, such as octahedron, six-helix bundle tubes or 24-helix bundle rods, were investigated using *in vitro* conditions, and time-and shape-dependent denaturation and digestion were observed due to the Mg^2+^ depletion in the media and the DNase activity of the serum [21]. As an alternative to the DNA origami method [14,15] and shape-specific designs, such as DNA cubes [22], tetrahedrons [23] or octahedrons [24], single-stranded tile assembly has recently proven to be a versatile and modular design strategy to build a wide variety of two- and three-dimensional shapes [25,26]. In this study, we intended to show efficient folate-mediated uptake and subsequent gene silencing by tile-assembled DNA nanotubes carrying GFP siRNAs *in vitro*. However, we were not able to demonstrate the sought-after effects, but instead observed untimely disassembly of our constructs under certain *in vitro* conditions and, therefore, investigated strategies to maintain the structural integrity in relevant environments. We examined the stability of tile-assembled structures under limited divalent cations and in the presence of nucleases in buffer and in cell media. We then describe a number of artifacts that should be taken into consideration during experiments with DNA-based nanostructures *in vitro*.

## 2. Results and Discussion

### 2.1. Design and Self-Assembly of Six-Helix DNA Nanotubes

**Scheme 1:**
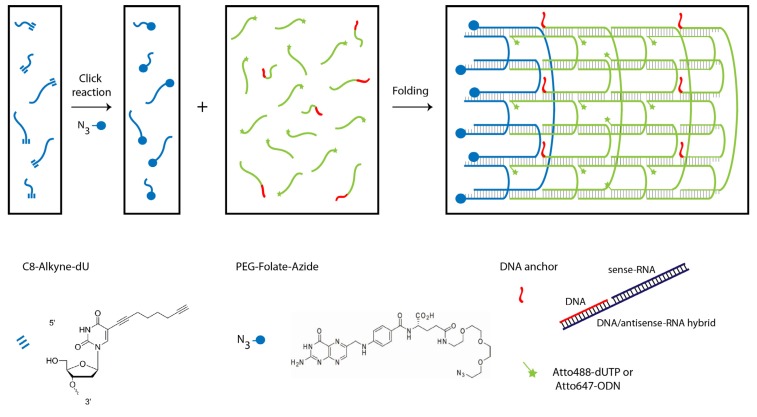
DNA nanotube assembly. (**Left**) Click reaction of alkyne-modified oligonucleotides with azide-modified PEGylated folate. (**Right**) Self-assembly of 24 oligonucleotides into a six-helix tube after a 17-h annealing process.

We designed tubule-like DNA nanostructures consisting of 24 oligonucleotides that self-assemble into six parallel helices using the single-stranded DNA tile assembly method introduced by Yin *et al.* (Scheme 1 and Table S1) [25,27]. Six of the oligonucleotides were alkyne-modified during synthesis and conjugated in-house with PEG-folate-azide (Baseclick GmbH, Tutzing, Germany) by a click reaction. Reversed phase high performance chromatography (RP-HPLC) analysis and matrix-assisted laser desorption/ionization (MALDI) mass spectrometry revealed the almost quantitatively conjugation of folate molecules to the alkyne-oligonucleotides. (Figures S1 and S2, Table S2). Another set of six oligonucleotides was extended by an 18 nt-long sequence at the 3' end to allow the attachment via hybridization of six siRNA molecules that potentially silence the expression of GFP upon delivery. To visualize the DNA nanotubes *in vitro*, two different labeling strategies were employed. In the first approach, Atto488-dUTP was enzymatically labeled to the 3' end of a set of 12 tile oligonucleotides using terminal transferase. In the second approach, the same set of oligonucleotides was extended with another 18 nt-long sequence allowing attachment via hybridization of 12 Atto647-modified (via NHS chemistry) oligonucleotides. The nanotubes have a designed length of ~27 nm and an expected diameter of ~6 nm for the dried sample. Note that the tube diameter of a six-helix bundle increases in buffer to 8 nm and that tubes decorated with additional molecules will have a larger effective diameter [28].

**Figure 1:**
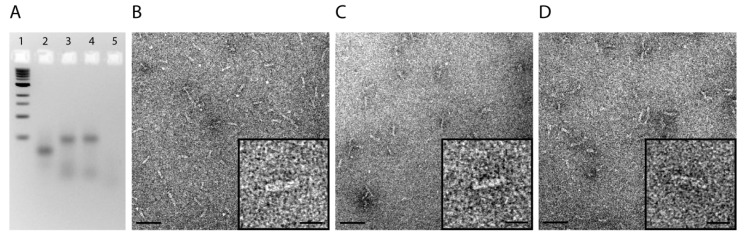
Characterization of nanotubes. (**a**) Gel electrophoresis analysis of assembled nanotubes: (1) 1-kb ladder, (2) nanotube, (3) nanotube + folate, (4) nanotube + folate + siRNA, and (5) individual oligonucleotide. Electron micrographs of (**b**) Nanotubes; (**c**) Nanotubes with folate; and (**d**) Nanotubes with folate and siRNA (scale bars: 50 nm; insets: 20 nm).

The nanotube structures containing the desired subsets of oligonucleotides and modifications were folded in TE-buffer containing 20 mM Mg^2+^ during a thermal annealing process starting at 80 °C and cooling down to room temperature over the course of 17 h. Analysis by gel electrophoresis analysis showed for all designs prominent bands representing the folded structures (Lanes 2 + 3 + 4 in Figure 1). Conjugation of folate and folate + siRNA (Lanes 3 and 4, respectively) to the DNA nanotubes leads to a decrease of their mobility in comparison to nanotubes without folate and siRNA (Lane 2). Transmission electron microscopy (TEM) demonstrates the correct assembly of the nanotubes and the monodispersity of the samples. The measured length of 27 ± 1 nm and the measured diameter of 6 ± 1 nm perfectly match the expected dimensions (Figure 1B–D).

### 2.2. Tubule-Like Tile-Assembled DNA Nanostructures Are Delivered to the Endosome of HeLa Cells Independently of Folic Acid and Are Not Capable of Releasing siRNA into the Cytosol

**Figure 2:**
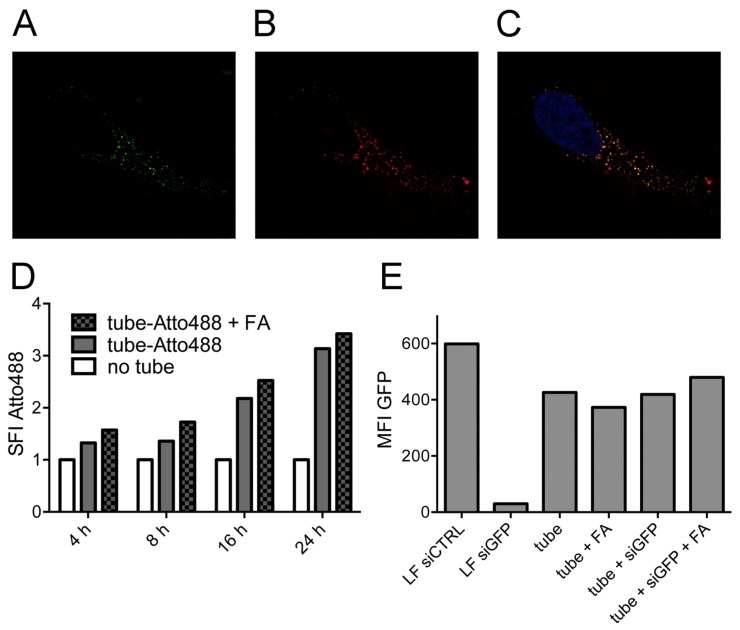
Endosomal uptake of nanotubes in HeLa cells. Endosomal staining of nanotubes with dextran. (**a**) Nanotubes; (**b**) Dextran; (**c**) Merged image from (**a**), (**b**) and a third channel (DAPI, blue). (**d**) Flow cytometry analysis of folate-dependent uptake of Atto488-labeled nanotubes over 24 h. Untreated cells act as the control, and the specific fluorescence intensity (SFI) of the dye is depicted. (**e**) Fluorescence intensity of stably GFP-expressing Hela cells upon the addition of nanotubes carrying *GFP*-targeting siRNAs or upon transfection of a *GFP*-targeting siRNA and a non-targeting siRNA, respectively, as controls using lipofection (LF). The median fluorescence intensity (MFI) of GFP is depicted.

DNA nanotubes labeled with Atto488 via enzymatic labeling were added to HeLa cell cultures at 10 nM, together with dextran-AF647 as a marker for endosomal uptake. At various time points thereafter, confocal microscopy was performed to evaluate the localization of the construct. After 24 h, we found clear co-localization of the nanotubes with dextran (Figure 2A–C). Observations for up to 72 h did not show any change in localization (Figure S3C,D).

To determine a potential effect of uptake via the folate receptor, which is highly expressed on the surface of HeLa cells, nanotubes with and without folic acid were compared side by side. No influence on the endosomal staining pattern was noticed in the fluorescence microscopy images, neither after 24 h nor after 72 h (Figure S3). For a quantitative analysis of the uptake, we conducted flow cytometry-based measurements of the HeLa cells at different time points after the addition of fluorophore-labeled nanotubes (Figure 2D). A minor signal was already detected after 4 h, which further increased in the course of 24 h. No significant difference was found between the uptake of nanotubes with or without folate.

On a functional level, we tested if the nanostructures released their siRNA cargo successfully to the cytoplasm by analyzing the knockdown capacity of siRNA molecules bound to the DNA nanotube. Stably *GFP*-transfected HeLa cell lines were used together with siRNA directed against *GFP* (siGFP). The siGFP was either bound to the nanostructure via hybridization or transfected into the cytoplasm by lipofection as a positive control. The GFP signal of the cells was measured by flow cytometry after 96 h (Figure 2E). In the condition with lipofection of GFP-targeting siRNAs, the fluorochrome signal was markedly decreased compared to lipofection of a control siRNA (siCTRL). However, the addition of siGFP to the nanotubes did not result in *GFP-*knockdown, independent of folate labeling, consistent with endosomal trapping of the whole structure, including their siRNA cargo.

### 2.3. Stability of DNA Nanotubes Differs in Various Conditions In Vitro

**Figure 3:**
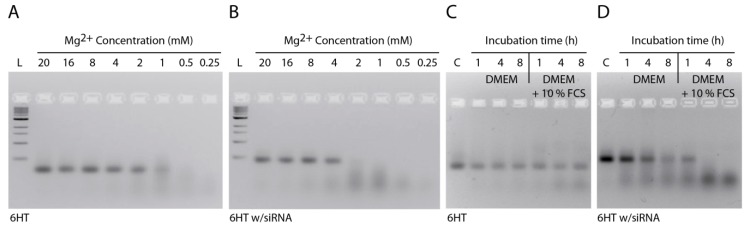
Stability of nanotubes. (**a**) Stability of nanotubes in PBS with different Mg^2+^ concentrations; (**b**) Stability of nanotubes carrying siRNA in PBS with different Mg^2+^ concentrations; (**c**) Stability of nanotubes in DMEM medium in the absence or presence of FCS; (**d**) Stability of nanotubes carrying siRNA in DMEM medium in the absence or presence of FCS (L: 1 kb ladder; C: control. All samples were incubated at 37 °C).

To address the stability of tile-assembled DNA nanostructures *in vitro*, we incubated them in different buffers and cell media. First, we incubated the nanotubes in PBS with different Mg^2+^ concentrations at 37 °C for 2 h. We used PBS as a buffer to simulate the cell media conditions, as both cell media and PBS possess several monovalent and divalent cations at isotonic concentrations. Importantly, for the assembly and stabilization of the DNA nanostructures, usually Mg^2+^ concentrations much higher than those found in PBS and cell media are used. While folding of DNA nanostructures can also be achieved at high Na^+^ concentrations [29], the 135 mM NaCl present in PBS are not sufficient to stabilize DNA nanotubes at 37 °C, if the individual DNA tiles are 42 nt long. Gel analysis revealed that the nanotubes without extensions were stable down to 1 mM Mg^2+^, whereas the nanotubes carrying siRNA started to degrade already below 4 mM Mg^2+^ (Figure 3A,B). This indicates that the addition of extension sequences protruding from the DNA nanotubes destabilizes the structure, which may be explained by distorted stacking of the last base before the extension and with an increase of electrostatic repulsion between the elongated tail and the DNA duplexes in the nanotube [30]. Next, we compared the stability of nanotubes against DNases and incubated the structures in cell medium containing 10% FCS. Gel analysis showed that under these conditions, the plain nanotubes are stable up to 8 h (Figure 3C). However, nanotubes carrying siRNA were degraded in 1 h when the structures were incubated in media containing 10% FCS. These nanotubes were also degraded slightly during 8 h in DMEM medium without FCS, likely due to Mg^2+^ depletion (in all cell media experiments, the concentration of Mg^2+^ was 1.8 mM).

**Figure 4:**
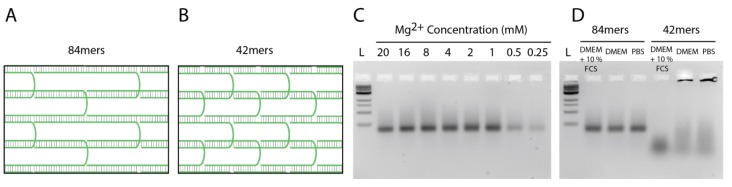
Stability of nanotubes assembled from 84 nt-long oligonucleotides. (**a**) Schematic depiction of a section of the 6HT demonstrating the hybridization of 84mers; (**b**) Schematic depiction of a section of the 6HT demonstrating the hybridization of 42mers; (**c**) Stability of nanotubes (84mers) in PBS with different Mg^2+^ concentrations; (**d**) Stability of nanotubes (84mers and 42mers) in DMEM + 10% FCS, DMEM and PBS. Nanotubes were incubated at 45 °C for 2 h (L: 1 kb ladder; C: control).

To overcome the problem of premature degradation, DNA tile tubes were assembled from 84 nt-long oligonucleotides. This design allows longer complementary regions (21 bp for the 84mers instead of 10 bp and 11 bp for the 42mers) within the tile assembly, which, in turn, yields much higher thermal stability, but also higher resistance to Mg^2+^ depletion (Figure 4). Our results show that the stability of tile-assembled nanotubes is dependent on sequence design, temperature, salt concentration and structural modifications, such as the addition of single- or double-stranded extensions to the DNA tiles.

### 2.4. Strong Extra-Endosomal Uptake Can Be Feigned by Dye Cleavage

**Figure 5:**
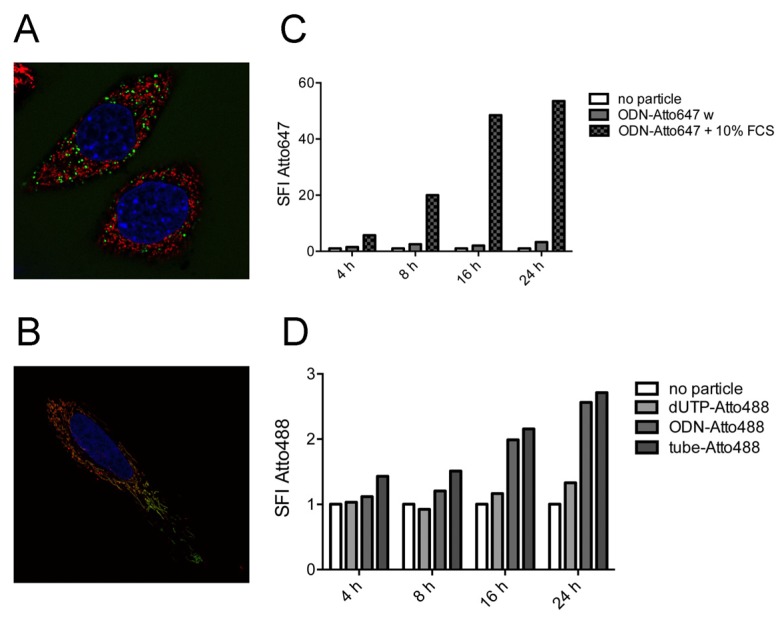
Effect of dye cleavage on cellular uptake. (**a**) Endosomal staining using Alexa Fluor 488-coupled dextran (shown in green) of HeLa cells treated with oligodeoxynucleotide (ODN)-Atto647 (shown in red); (**b**) Mitochondrial colocalization of Atto647 (shown in red) in HeLa cells stained with the mitochondrial dye MitoTracker Green (shown in green). Nuclei are stained with Hoechst 33342; (**c**) Flow cytometry analysis of fluorescence intensity of cells treated with ODN-Atto647 in the absence or presence of FCS; (**d**) Flow cytometry analysis of fluorescence intensity of cells treated with Atto488-dUTP, ODN-Atto488 and nanotube labeled with Atto488. Untreated cells act as the control, and the specific fluorescence intensity (SFI) of the dye is depicted.

When nanostructures labeled with Atto647 via hybridization were incubated with HeLa cells, we repeatedly observed a very high fluorescence level in the cells during microscopy- or flow cytometry-based analysis. Furthermore, the fluorochrome did not co-localize with dextran as an endosomal marker (Figure 5A), but instead, mitochondrial localization was detected (Figure 5B). The level of uptake and the mitochondrial staining pattern were associated with the addition of serum to the culture medium (Figure S4). Similarly, when only the oligonucleotide labeled with Atto647 (via NHS chemistry) was added to the HeLa cells, we observed a rapid and strong staining of the cells only in the case when serum was added (Figure 5C). This effect was not observed when the fluorophores were attached via enzymatic binding. We therefore conclude that Atto647 is cleaved off the DNA by some component in the serum and is taken up independently of the nanostructure.

### 2.5. Single-Stranded DNA Molecules, But Not Deoxynucleotide Triphosphates, Are Internalized at Similar Levels as the Tile-Assembled Nanotube Structures

Specific uptake of the tubule-like tile-assembled DNA nanostructures was analyzed by direct comparison with oligonucleotides and deoxynucleotide triphosphates. All three molecules were labeled with Atto488 and incubated at identical molar concentrations with HeLa cells. Fluorochrome uptake was measured by flow cytometry at various time points (Figure 5D). No intracellular staining was found in the deoxynucleotide triphosphate condition. However, we observed similar uptake of the fluorochrome with the oligonucleotide as with the nanostructure.

## 3. Experimental Section

### 3.1. DNA Nanotube Design

DNA nanotubes were designed using Yin’s single-strand tile (SST) method [27]. Each tile oligonucleotide is 42 bases long and consists of four domains with ten or eleven bases. Twenty four individual oligonucleotides were used to form 6 helix nanotube. The domains at the ends of the nanotube contain non-pairing poly-A sequences to prevent polymerization. siRNA hybridization to the nanotubes was done by extending 3' ends of six tiles with an 18-nt long overhang sequence (5'-AGGATGTAGGTGGTAGAG-3'). The used siRNA sequences for *GFP* silencing were sense: 5'-GCCACAACGUCUAUAUCAU-3', and antisense: 5'-AUGAUAUAGACGUUGUGGC CTCTACCACCTACATCCT-3'. Six oligonucleotides were modified with PEG-folate azide (Baseclick GmbH, Tutzing, Germany) using click reactions. The underlined sequence shows the complementary overhang. All oligonucleotides were purchased from Eurofins (Ebersberg, Germany) with HPSF or HPLC purification.

### 3.2. Folate Conjugation and Characterization of Oligonucleotides

Each of the six alkyne-modified oligonucleotides (Baseclick GmbH) were submitted to click reaction, using CuBr as the Cu(I) source. Ten microliters of a freshly prepared CuBr (0.1 M)/THPTA (0.1 M) solution in a 1:2 *v*/*v* ratio were added to a 50-µL (0.1 mM, 5 nmol) solution of each alkyne-oligonucleotide. The addition of folate-PEG3-azide (2.5 µL, 10 mM in DMSO) completed the click reaction cocktail. The mixture was mixed for 1.5 h at 45 °C. Finally, the solution was purified via ethanol precipitation. Folate-conjugated oligonucleotides were analyzed by analytical RP-HPLC (e2695 system, Waters, Milford, MA, USA) coupled with a photodiode array detector (PDA 2998, Waters) using a reversed phase XBridge OST C18 column (4.6 mm × 50 mm, Waters). Before injection in RP-HPLC, samples were diluted to 10 µM concentration in HPLC-grade H_2_O. Samples (2 µL) were desalted against ddH_2_O using a nitrocellulose membrane (MerckMillipore, Frankfurt, Germany), and 0.4 µL were spotted onto a MALDI plate (Bruker Corporation, Millerica, MA, USA) along with 0.4 µL of 3-hydroxypicolinic acid (HPA, Sigma Aldrich, St. Luis, MO, USA). Measurements were carried out with the Autoflex MALDI-TOF (Bruker Corporation).

### 3.3. Dye Labeling of DNA Nanotubes

Fluorescent dyes were conjugated to the 12 oligonucleotides with two different approaches before the nanotube assembly. In the first approach, 3' ends of twelve tiles were extended by 18 nt-long overhang sequences to hybridize the dye-modified sequence: (Atto647 TTCATTCTCCTATTACTACC). In the second approach, the 3' ends of the same tiles were enzymatically labeled with Atto488-dUTP (Jena Bioscience, Jena, Germany). For this, Atto488-dUTP (80 µM), CoCl_2_ (5 mM), terminal transferase enzyme (16 U/µL, Roche, Penzberg, Germany) and all DNA tiles (400 pmol) were mixed in a 20 µL, 1× TdT reaction buffer and then incubated at 37 °C for 30 min. Then, 2.5 µL of NaOAc (3 M) were added, and the solution was filled up to 80 µL with ice-cooled ethanol (99%). After 1 h of incubation at −20 °C, samples were centrifuged at 13,000× *g* for 30 min. Then, samples were washed with 70% ethanol for 10 min again, and the supernatant was discarded. The remaining pellet was dissolved in distilled water.

### 3.4. DNA Nanotube Assembly and Purification

For DNA nanotube assembly, 400 nM of dye and folate modified tiles, 800 nM of antisense-ODN and 1.6 µM of sense-ODN were mixed in a folding buffer (10 mM Tris-HCl, 1 mM EDTA, pH 8.0, 20 mM MgCl_2_). For the plain nanotube assembly, 1 µM of unmodified tiles was used. The DNA nanotubes were folded over the course of 16 h (5 min at 80 °C, cooling down to 65 °C at 1 °C/min, cooling down to 25 °C at 2.5 °C/h). Purification of the assembled DNA nanotubes was done using 30K Amicon Ultra 0.5-mL centrifuge filters (30000 MWCO, Millipore, Schwalbach, Germany) to remove excess strands that were not folded into the structures. One hundred microliters of assembled DNA nanotube solution were mixed with 400 µL of folding buffer, filled into the centrifuge filter, and centrifuged 3 times at 13,000× *g* for 6 min. After every centrifuge step, the flow-through was removed and the filter was refilled up to 500 µL with buffer. After final centrifugation, the remaining solution at the bottom of the filter (~50 µL) was pipetted out, and the concentration of nanotubes was determined by measuring the optical density at 260 nm.

### 3.5. Gel Electrophoresis and Transmission Electron Microscopy

DNA nanotubes were analyzed by running samples in an agarose gel. For this, 2% agarose was dissolved in 0.5× TAE buffer by heating to boiling. MgCl_2_ (11 mM) and ethidium bromide (0.5 µg/mL) were added after the cooling, and the solution was poured into a gel cask for solidification. Twenty microliters of each filter-purified DNA nanotube sample were mixed with 4 µL of 6× loading dye before loading into the gel pockets. Six microliters of 1 kb ladder were also loaded adjacent to the samples. The gel was run for 2 h at 70 V in an ice-cold water bath to prevent heat-induced denaturation of DNA nanotubes. To visualize DNA nanotubes, a JEM-1011 transmission electron microscope (JEOL GmbH, Eching, Germany) was used. DNA nanotubes were incubated on plasma-exposed (240 kV for 1 min) carbon-coated grids and then negatively stained with 2% uranyl formate for 10 s.

### 3.6. Stability of DNA Nanotubes

The stability of DNA nanotubes was tested in PBS buffer, DMEM and DMEM containing 10% FCS, separately. One microliter of DNA nanotubes (50 ng/µL) in 20 µL of buffer/medium was incubated at 37 °C for different time points. Two percent agarose gel with 11 mM MgCl_2_ was prepared to analyze the samples, as mentioned in Experimental Section 3.5.

### 3.7. Cell Culture Experiments

HeLa cells were cultured at 37 °C, 5% CO_2_ and 95% humidity in Dulbecco’s modified Eagle’s medium (Life Technologies Thermo Fischer, Waltham, MA, USA) supplemented with 10% heat-inactivated fetal calf serum (FCS, Invitrogen Thermo Fischer, Waltham, MA, USA), 2 mM L-glutamine, 100 U/mL penicillin and 100 µg/mL streptomycin. Stably GFP-transfected HeLa cell lines were generated by retroviral transduction using an eGFP containing pMP71 vector, as described previously [31,32]. To analyze uptake, DNA nanotubes, oligonucleotides and deoxynucleotide triphosphates were added to HeLa cells at a concentration between 10 and 40 nM for the indicated period of time in DMEM with L-glutamine, penicillin, streptomycin and either supplemented or not with 10% FCS. For siRNA-mediated knockdown experiments, GFP-expressing HeLa cells were seeded in 24-well plates and allowed to adhere overnight. On the next day, cells were either incubated with the indicated nanotubes coupled to GFP-targeting siRNAs or were transfected as a control with siRNA oligonucleotides (75 nM) using Lipofectamine RNAiMAX (Invitrogen). After 48 h, GFP-knockdown was measured by flow cytometry. The siRNA sequence used to target GFP (siGFP) was 5' GCCACAACGUCUAUAUCAU 3'. As a control (siCTRL), we used the non-targeting RNA sequence 5' GCGCUAUCCAGCUUACGUA 3' described previously [33]. SiRNAs were purchased from Eurofins and contained dTdT overhangs.

### 3.8. Flow Cytometry and Confocal Fluorescence Microscopy

Flow cytometry was used to determine the uptake of DNA nanotubes, oligonucleotides and deoxynucleotide triphosphates labeled with the fluorescent dyes, Atto488 or Atto647, into HeLa cells and to assess the knockdown efficiency of *GFP*-targeting siRNAs in stably GFP-expressing HeLa cells. For that, after the indicated time points, single-cell suspensions were prepared and washed several times before analyzing the cells on a FACS Calibur (Becton Dickinson, Franklin Lakes, NJ, USA). FlowJo software was used to analyze the data. GFP expression was depicted by median fluorescence intensity (MFI). For all experiments with fluorescent dye labeling, the data are represented as specific fluorescence intensity (SFI), which was calculated by dividing the MFI of the sample by the MFI of the control. Confocal fluorescence microscopy was used to determine the subcellular localization of nanotubes and RNAs taken up by HeLa cells. For that, HeLa cells were cultured in CELLview cell culture dishes with a glass bottom (Greiner Bio One). After incubation with Atto488- or Atto647-labeled nanotubes for the indicated time points, cells were washed three times with PBS and used for live-imaging on a Leica TCS SP5 confocal microscope (Leica Microsystems GmbH, Wetzlar, Germany). Zero-point-two micrograms per milliliter of Hoechst 33342 and MitoTracker Green (both from Life Technologies) were used according to the manufacturer’s protocol to stain nuclei and mitochondria, respectively. In order to visualize endosomes, 20 µg/mL dextran labeled with Alexa Fluor 647 or Alexa Fluor 488 (Life Technologies Thermo Fischer) were added simultaneously with the DNA nanotubes.

## 4. Conclusions

In this study, we investigated the cellular delivery of tile-assembled DNA nanotubes carrying siRNAs using GFP-expressing HeLa cells via folate targeting. We observed that the nanostructures enter the cells via an endosomal pathway, but the nanostructures and their siRNA cargo are not capable of reaching the cytoplasm for knockdown and gene silencing. Contingently, no significant decrease in GFP expression levels was detectable, and folate modification did not change the uptake kinetics. The stability experiments revealed that unmodified DNA nanotubes are stable at 37 °C up to 8 h in the cell media and that they stay intact in PBS buffer containing 2 mM Mg^2+^ or more. However, the extension of the DNA tile strands with sequences that allow the hybridization of siRNA or dye-modified strands drastically decreases the construct’s stability, a fact that may have contributed to the unsuccessful folate targeting experiments. Using DNA tiles that were 84-nt long drastically increased the stability in all cell media and buffers with low Mg^2+^ concentrations. Importantly, we observed that DNA strands alone and cleaved dyes are also uptaken by the cells, which can lead to the misinterpretation of recorded data. Overall, the results presented in this study demonstrate the importance of rigorously testing the stability of DNA nanostructures before applications *in vitro* and *in vivo*.

## Supplementary Materials

Supplementary materials can be accessed at: http://www.mdpi.com/2079-4991/5/1/47/s1.
