# Supplementary material for: Cellular Uptake of Tile-Assembled DNA Nanotubes
Source: Nanomaterials (Basel). 2014 Dec 30;5(1):47–60. doi: 10.3390/nano5010047 (PMC5312849; doi:10.3390/nano5010047)
Supplement: Supplementary file 1 [file nanomaterials-05-00047-s001.pdf]

Supporting Information

Table S1. The list of oligonucleotide sequences used in the 6-helix tile-tube assembly.

(**A**) Oligonucleotides for folate conjugation. X: C8-Alkyne-dU.

ODN-1	XAAAACGCTAAGCCACCTTTAGATCCAAA
ODN-2	XGGATCTAAAGGACTTCTATCAAAGACGGGACGACTCCGGGAG
ODN-3	XAAACTCCCGGAGTCCGCTGCTGATCAAA
ODN-4	XGATCAGCAGCGCCCGTCTCGACTGCAGAAATAGGACCCCCAG
ODN-5	XAAACTGGGGGTCCTCGAGGCGAAACAAA
ODN-6	XGTTTCGCCTCGTAGCCTTCGCCCGCACGACCTGGCTTAGCGT

(**B**) Oligonucleotides for fluorescent dye labeling. Z: Atto488-dUTP or overhang sequence for Atto647 modified oligonucleotide (GGTAGTAATAGGAGAATG).

GGTCGTGCGGACTGTCGAACACCAACGATGCCTGATAGAAGTZ
GCGTGGCAATTGCCATAAATTCATACATAACGGCGCCAGACGZ
TTTCAAGACCGGCACTTGTATGGCGTAGGGCGGGTTTAGCGGZ
CGTTATGTATGCCGCTAAACCTTGCAATGACTGAACTCGAACZ
GTCCCGTCTTTGGATCCGAAAGCCATAATATATCGAGACGGGZ
TCGAAGTCGTGTTCGAGTTCAAATGTCTATGCGATGCAGCAGZ
GTCATTGCAATAGCTCCCATCATTTAATGTCGTTTACAGTAAZ
GCATAGACATTTTACTGTAAAACCTTACGTAACTTACAGCCAZ
ATTTCTGCAGGGAATTCAGCCTATTCACATAGGCGAAGGCTAZ
ATGCCAGGAATGGCTGTAAGTTGCATCATGGGGGTCCTCAATZ
TACGTAAGGTCAATACTCATCCCTGAGTGATCCATGACCCTTZ
CCCATGATGCAAAGGGTCATGGGTCTTGAAAAATTTATGGCAZ

(**C**) Oligonucleotides for siRNA labeling.

GGCATCGTTGGCGTCTGGCGCACGACTTCGATTTCGGATCCAAGGATGTAGGTGGTAGAG
CGCCCTACGCCAAAAAAGATGGGAGCTAAGGATGTAGGTGGTAGAG
TATATTATGGCCTGCTGCATCTTCCTGGCATGGCTGAATTCCAGGATGTAGGTGGTAGAG
CGACATTAAATAAAAAAGATGAGTATTGAGGATGTAGGTGGTAGAG
CTATGTGAATAATTGAGGACCATTGCCACGCTGTTCGACAGTAGGATGTAGGTGGTAGAG
GATCACTCAGGAAAAAAATACAAGTGCCAGGATGTAGGTGGTAGAG

Table S2. Molecular weights of 6 oligonucleotides before and after folate conjugation.

**Samples**	**MW (g/mol)**		**MW (g/mol) after reaction**	
	**expected**	**experimental**	**expected**	**experimental**
ODN-1	8913	8920,382	9554,64	9547,765
ODN-2	14355	14602,380	14996,64	14992,571
ODN-3	8937	8931,952	9578,64	9574,670
ODN-4	14197	14195,535	14838,64	14842,370
ODN-5	9075	9071,732	9716,64	9710,265
ODN-6	14119	14142,973	14760,64	14759,459
Folate-PEG3-Azide	641,64			

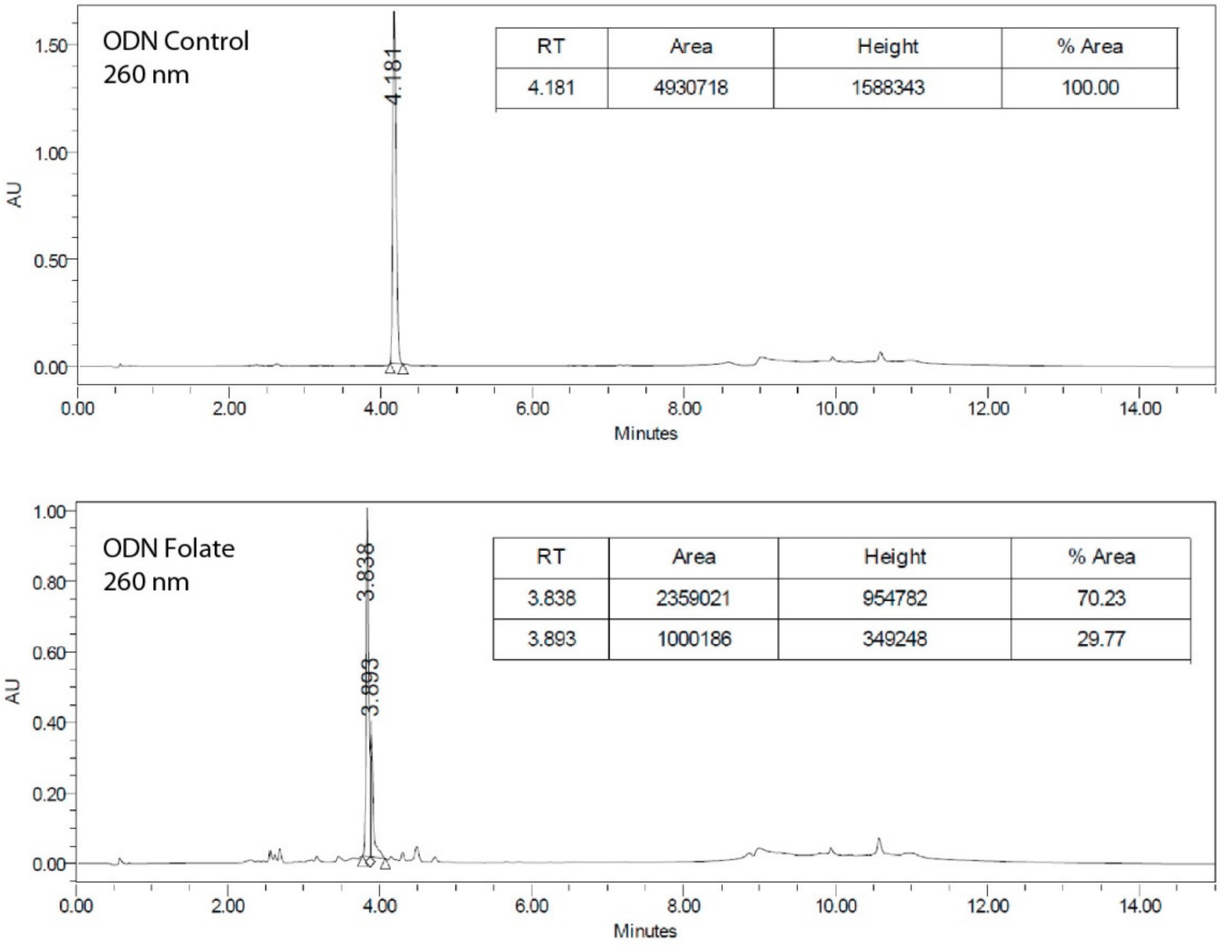

Figure S1. RP-HPLC chromotogram of a single DNA oligonucleotide before (upper chromatogram) and after (bottom chromatogram) folate conjugation.

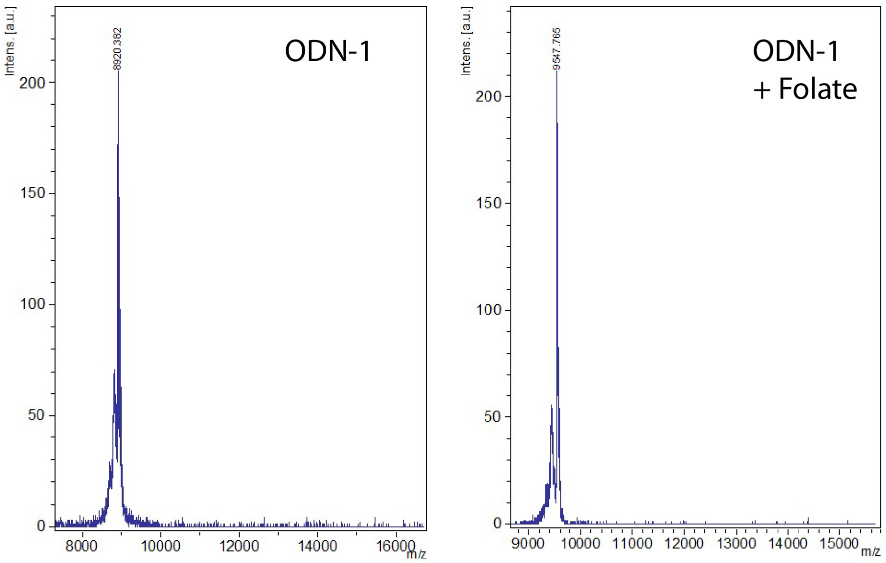

Figure S2. *Cont.*

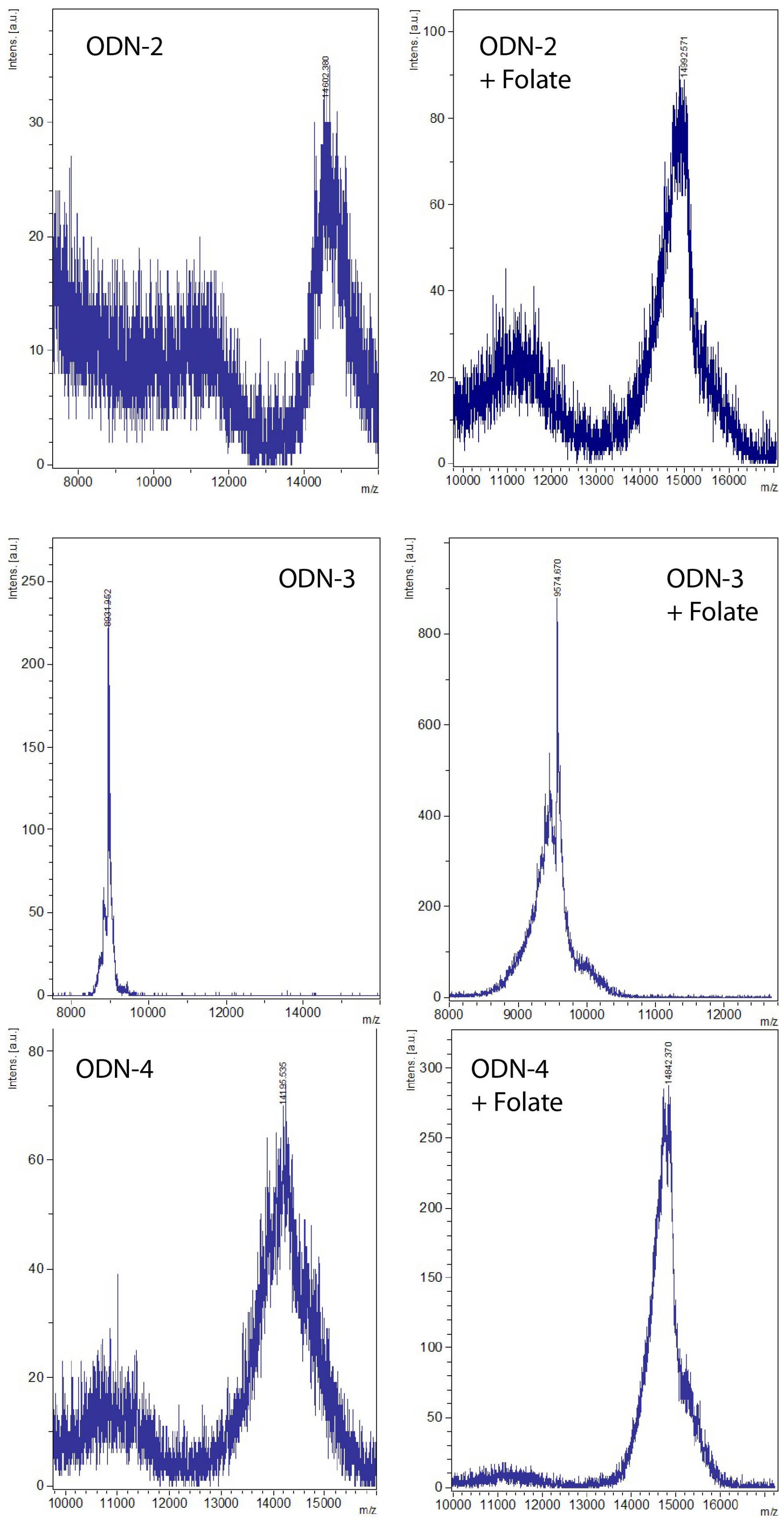

Figure S2. *Cont.*

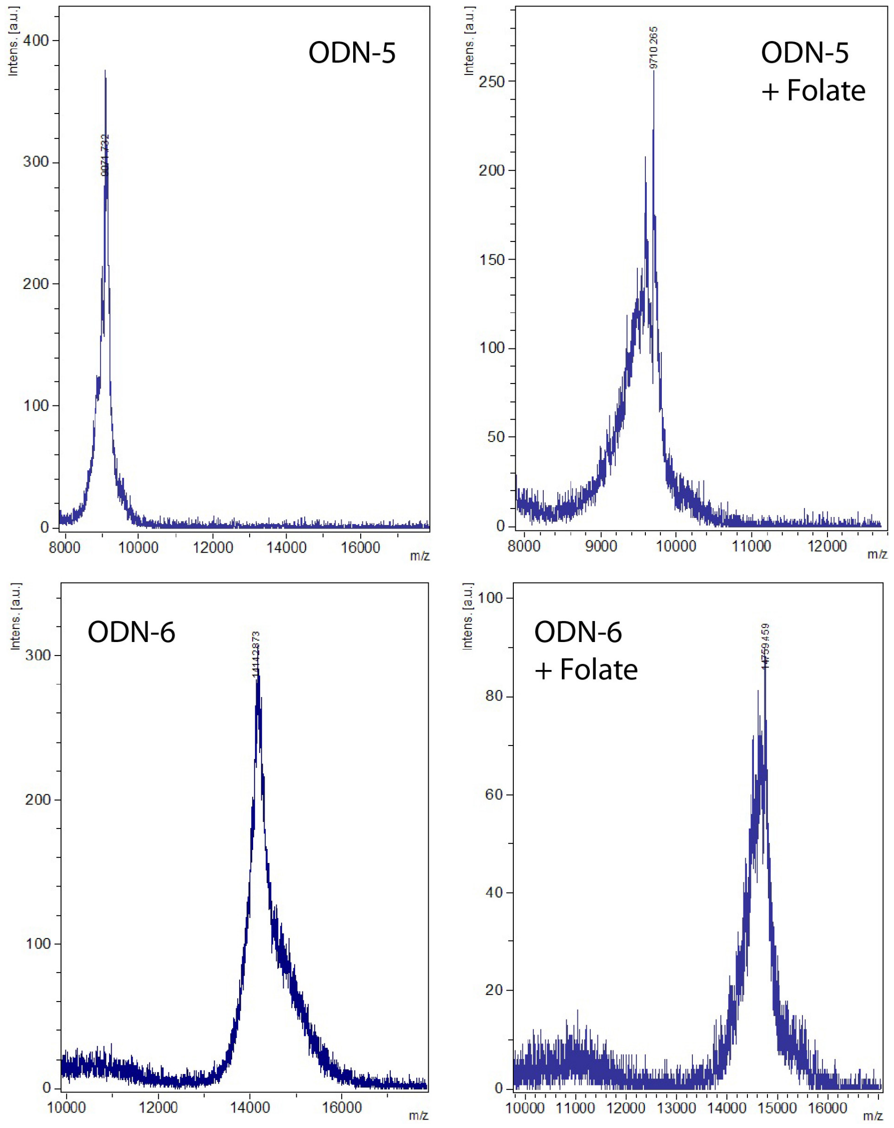

Figure S2. Mass spectrometry analysis of 6 oligonucleotides before and after folate conjugation.

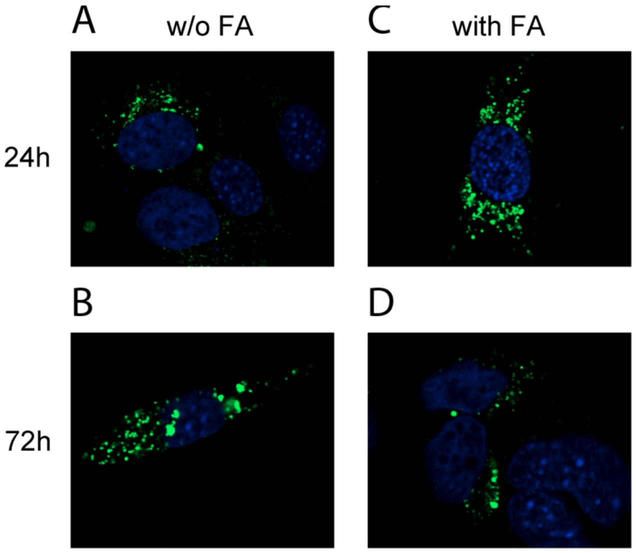

Figure S3. Endosomal uptake of unmodified (**a**,**b**) and folate-modified (**c**,**d**) DNA nanotubes after 24 h and 72 h of incubation.

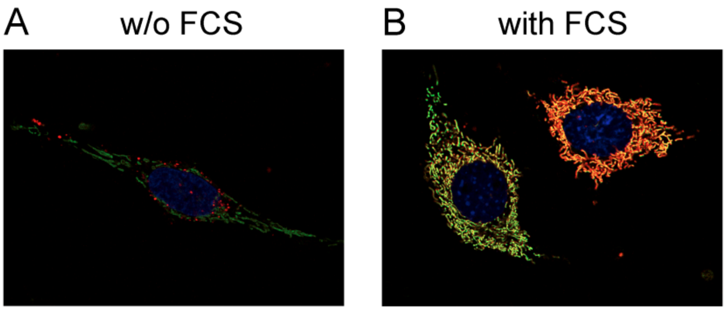

Figure S4. Mitochondrial colocalization of the mitochondrial dye Mito-tracker green (shown in green) and DNA nanotubes coupled to Atto 647 (shown in Red) after culture medium (**a**) without FCS and (**b**) with FCS.

© 2014 by the authors; licensee MDPI, Basel, Switzerland. This article is an open access article distributed under the terms and conditions of the Creative Commons Attribution license (http://creativecommons.org/licenses/by/4.0/).
